# Different antithrombotic strategies after coronary artery bypass grafting to prevent adverse events: a retrospective analysis

**DOI:** 10.1186/s13019-024-02937-y

**Published:** 2024-07-04

**Authors:** Yi Shi, Shenglong Chen, Gang Liu, Bo Lian, Yu Chen, Lixue Zhang

**Affiliations:** 1 Department of Cardiac Surgery, Peking University People’s Hospital, Peking University Health and Science Center, Xizhimen St, Beijing, 100044 China; 2 Cardiac Center, Anhui Second People’s Hospital, Hefei City, Anhui China

**Keywords:** Antithrombotic strategies, Coronary artery bypass grafting, Dual antiplatelet therapy

## Abstract

**Objective:**

Coronary artery bypass grafting (CABG) is associated with antithrombotic therapy in terms of postoperative adverse events; however, it is still unknown whether the early use of such drugs after CABG is safe and effective. In this study, we aim to evaluate the relationship between different postoperative antithrombotic strategies and in-hospital adverse events in patients undergoing isolated coronary artery bypass grafting surgery.

**Methods:**

This was a single-center, retrospective cohort analysis of patients undergoing isolated CABG due to coronary artery disease (CAD) between 2001 and 2012. Data were extracted from the Medical Information Mart for Intensive Care III database. The patients involved were divided into the ASA (aspirin 81 mg per day only) or DAPT (aspirin plus clopidogrel 75 mg per day) group according to the antiplatelet strategy. Patients were also stratified into subgroups based on the type of anticoagulation. The in-hospital risk of bleeding and adverse events was investigated and compared between groups. Propensity score matching (PSM) was performed to reduce the potential effects of a selection bias.

**Results:**

A total of 3274 patients were included in this study, with 2358 in the ASA group and 889 in the DAPT group. Following the PSM, no significant difference was seen in the risk of major bleeding between the two groups according to the PLATO, TIMI or GUSTO criteria. There was no difference in the postoperative mortality. In subgroup analysis, patients given anticoagulant therapy had an increased incidence of bleeding-related events. Multivariable analysis revealed that postoperative anticoagulant therapy and the early use of heparin, but not DAPT, were independent predictors of bleeding-related events.

**Conclusions:**

Postoperative DAPT was not associated with an increased occurrence of bleeding-related events in patients undergoing isolated CABG and appears to be a safe antiplatelet therapy. The addition of anticoagulants to antiplatelet therapy increased the risk of bleeding and should be considered cautiously in clinical practice.

## Introduction

Coronary artery bypass grafting surgery (CABG) is a major revascularization approach for patients with coronary artery disease (CAD) and is widely used across the world. According to the current guidelines, CABG is recommended for stable CAD with proximal left anterior descending stenosis, left main stenosis and three-vessel disease, especially when the patient has a high SYNTAX score or diabetes mellitus [1]. CABG is performed to relieve the symptoms of myocardial ischemia and improve the prognosis. Perioperative management is critical to reduce the main adverse cardiovascular and cerebrovascular events and complications after surgery.

Antithrombotic treatment plays an important role in reducing adverse cardiovascular events and is mandatory in CAD patients undergoing myocardial revascularization [2]. Antiplatelet therapy is recommended early after operation for patients with low bleeding risk [2] due to its prevention of early graft failure as well as its reduction of adverse events and death after CABG in some studies [3–5]. Early use of dual antiplatelet drugs is also reasonable for patients with a high risk of ischemia and thrombosis, but the risk of bleeding should be taken into consideration [2]. In addition, heparin for anticoagulation is administered in some centers at an early stage after CABG for the prevention of ischemic events [6]. However, the current guidelines make no recommendation on the early use of heparin after CABG. Whether it is safe and effective to prevent early adverse events is still unknown.

## Materials and methods

### Database introduction

This was a single-center, retrospective cohort analysis of patients undergoing isolated CABG between 2001 and 2012. Data were extracted from the Medical Information Mart for Intensive Care III (MIMIC III, V1.4) database, which is maintained by the Laboratory for Computational Physiology at the Massachusetts Institute of Technology. It contains information on more than 40,000 patients in the ICU at Beth Israel Deaconess Medical Center. The database is accessible to researchers who have completed “protecting human subjects” training. The institutional review boards of the Massachusetts Institute of Technology (Cambridge, MA) and Beth Israel Deaconess Medical Center (Boston, MA) approved the establishment of the database. Thus, consent was obtained for this study. Data presented in this study were extracted by author Zhang, who completed the online training course of the National Institutes of Health (certification number: 35,943,280). Data extraction was performed using PostgreSQL tools V.1.12.3.

### Study population

Patients undergone isolated CABG due to CAD between 2001 and 2012 were selected from the database. Perioperative administration of antithrombotic therapy was extracted, including the types of drugs and the duration of usage. The following information were: age; sex; weight; smoking history; comorbidity; diagnosis of acute coronary syndrome (ACS); history of PCI; history of CABG; type of operation; preoperative level of creatinine and platelet count; perioperative level of hemoglobin; postoperative mortality during hospitalization; postoperative MI, stroke and bleeding during hospitalization; postoperative transfusion of packed red blood cells (PRBCs), fresh frozen plasma (FFP) and platelets; and postoperative mediastinal chest tube drainage (MCTD). Duplicated records and records with missing information on antithrombotic therapy were excluded. Besides, patients with aspirin or clopidogrel contraindications, metal valve, the presence of persistent atrial fibrillation preoperatively, incarceration, or refusal to follow-up were excluded from the study.

### Study (sub)groups

Patients were divided into subgroups based on the type of postoperative antithrombotic therapy. The main study groups were defined as follows:

1. ASA group (single anti-platelet therapy with aspirin 81 mg per day postoperatively).
  - ASA non-anticoagulation subgroup (aspirin without anticoagulative therapy postoperatively).
  - ASA anticoagulation subgroup (aspirin + anticoagulative therapy postoperatively).
2. DAPT group (dual anti-platelet therapy of aspirin 81 mg per day + clopidogrel 75 mg per day postoperatively).
  - DAPT non-anticoagulation subgroup (aspirin + clopidogrel without anticoagulative therapy postoperatively).
  - DAPT anticoagulation subgroup (aspirin + clopidogrel + anticoagulative therapy postoperatively).

Anti-platelet therapy was used after the weaning of mechanical ventilation. Anticoagulative therapy included heparin, low-molecular-weight heparin (LMWH), and warfarin alone and their combinations. Patients were also grouped by the early use of heparin postoperatively (within 48 h after surgery) or not. Drug type, dose and duration were collected from the database.

### Definitions and outcomes

The primary endpoint was postoperative bleeding events during hospitalization according to the PLATO, TIMI and GUSTO criteria. In the PLATO criteria [7], major life-threatening bleeding was defined as fatal bleeding, intracranial bleeding, intrapericardial bleeding with cardiac tamponade, hypovolemic shock or severe hypotension due to bleeding requiring pressors or surgery, a fall in hemoglobin of 50 g/L (3.1 mmol/L) or greater, or the need for transfusion of at least 4 units of red blood cells. Other major bleeding was defined as significantly disabling bleeding (such as intraocular bleeding with permanent vision loss), a drop in hemoglobin of at least 30 g/L (1.9 mmol/L) but < 50 g/L (3.1 mmol/L) or requiring transfusion of 2 to 3 units of red blood cells. The TIMI criterion for major bleeding was either intracranial hemorrhage or bleeding associated with a decrease in hemoglobin concentration of more than 50 g/L [8]. Severe bleeding in the GUSTO guideline was defined as fatal intracranial intrapericardial bleeding with cardiac tamponade, or the development of hypovolemic shock or severe hypotension due to bleeding requiring pressor support or surgery [9]. Secondary endpoints included postoperative mortality, postoperative myocardial infarction (MI), and postoperative stroke during hospitalization, and the composite of mortality, MI and stroke. Postoperative MI was defined in accordance with the fourth universal definition of myocardial infarction [10]. Other secondary endpoints included PRBC (packed red blood cell), FFP (fresh frozen plasma) or platelet transfusion, and MCTD (mediastinal chest tube drainage) > 1 L within 12 h postoperatively. According to the criteria proposed by The Bleeding Academic Research Consortium (BARC) which classified bleeding events based on bleeding cause, site, severity, and outcomes into 5 types, CABG-related bleeding falls on Type 4 and is divided into the following categories: (1) perioperative intracranial bleeding within 48 h, (2) reoperation after closure of sternotomy for the purpose of controlling bleeding, (3) transfusion of ≥ 5 units of whole blood or packed red blood cells within a 48-hour period, (4) chest tube output ≥ 2 L within a 24-hour period [11].

### Statistical analysis

Propensity score matching (PSM) was performed to reduce the potential effects of a selection bias. The PSM was carried out using the nearest neighbor method on a logit scale (ratio = 1:1; caliper width = 0.05). Continuous variables are presented in the tables as the mean with SD or median with interquartile ranges. Student’s t-test or the Mann-Whitney U-test was used as appropriate. Categorical variables are presented as percentages and were compared using the χ^2^ test or Fisher’s exact test. Multivariable binary logistic regression analysis with an enter selection method was performed to determine variables predictive of bleeding-related events and postoperative adverse events. Goodness of fit was assessed for all logistic regression models. A *P*-value of < 0.05 was considered statistically significant. Statistical analysis was performed using SPSS Statistics 25 (IBM Corp., Armonk, NY, USA).

## Results

### Population and baseline characteristics

The MIMIC III database contains records of 9243 patients undergoing isolated CABG due to CAD between 2001 and 2012. Patients were excluded if they were duplicated or unrecorded, or they had contraindications to either aspirin or clopidogrel, declined to be followed-up or were incarcerated. We also excluded the patients with metal valve or the presence of atrial fibrillation since anticoagulant therapy is necessary in this population. Moreover, we included patients that undergone emergent CABG, since this population usually is under-presented in registries and are more likely to receive DAPT. Therefore, in the end, a total of 3274 patients were included in this study. Of these, 72.8% (*n* = 2385) were treated with ASA alone postoperatively, and the rest (*n* = 889) were treated with ASA and clopidogrel. Moreover, 33.3% of the patients also received anticoagulant therapy after the surgery in the ASA (*n* = 794) and DAPT (*n* = 296) groups. Patients at high risk of thrombosis (e.g., ACS or coronary stenting within 30 days) who have had a minor/major bleeding event are advised to continue low-dose aspirin. Early reactivation of a second antiplatelet agent should be considered after bleeding control. The use of perioperative antiplatelet therapy was decided by individual surgeons. Clopidogrel should be stopped at least 5 days before surgery. There were no specific rules or restrictions from local authorities on selecting patients for post-CABG DAPT. Typically, aspirin 100 mg daily was started within 24 h (ideally within 6 h) after CABG and recommended to continue long-term. For DAPT, 75 mg clopidogrel daily was added to 100 mg aspirin without a loading dose, preferably within 48 h after CABG once clinical stability was ensured and chest tube output was < 30 mL/hour for at least 2 h. The duration of DAPT was determined by the treating physician, with a minimum of 1 month. Proton-pump inhibitors were recommended as a prophylactic therapy for gastrointestinal bleeding. Additional therapies like statins, beta-blockers, or renin-angiotensin system inhibitors were recommended for secondary prevention in suitable patients, following clinical guidelines. In summary, the choice of perioperative antiplatelet therapy was up to individual surgeons. Post-CABG, aspirin was started quickly and continued indefinitely. Clopidogrel could be added for DAPT after ensuring clinical stability, for a minimum of 1 month as decided by physicians. Other secondary prevention medicines were used as suitable per guidelines. The baseline characteristics of patients in the ASA and DAPT groups are presented in Table 1. Before matching, the two groups were balanced regarding most comorbidities, preoperative ASA and heparin use, and postoperative anticoagulant therapy which were determined by claimed prescriptions after surgery to reduce ischemic events. The DAPT group had a higher proportion of patients with peripheral artery disease, ACS, previous PCI or previous CABG history. Additionally, lower preoperative hemoglobin values and higher preoperative platelet counts were seen in the DAPT group. Following PSM, the 2 groups (*n* = 868) were balanced regarding all the baseline characteristics.

**Table 1:** The baseline characteristics of patients in the ASA and DAPT groups

	before PSM			After PSM		
	ASA group (*n* = 2385)	DAPT group (*n* = 889)	*p*-value	ASA group (*n* = 868)	DAPT group (*n* = 868)	*p*-value
Age (years)	67.3 (59.7–74.7)	66.5 (58.5–74.0)	0.044	66.6 (59.0-74.5)	66.7 (58.6–74.1)	0.684
Male	1877 (78.7)	661 (74.4)	0.008	665 (76.6)	646 (74.4)	0.289
Weight (kg)	87.8 (77.5–97.0)	87.6 (76.8–96.6)	0.437	87.9 (78.0–97.0)	87.7 (76.8–96.6)	0.551
Smoke	246 (10.3)	87 (9.8)	0.657	93 (10.7)	85 (9.8)	0.527
Hypertension	1650 (69.2)	604 (67.9)	0.495	592 (68.2)	592 (68.2)	1.000
DM	963 (40.4)	366 (41.2)	0.681	389 (44.8)	356 (41.0)	0.110
Dyslipidemia	1618 (67.8)	574 (64.6)	0.077	585 (67.4)	557 (64.2)	0.157
Peripheral artery disease	96 (4.0)	81 (9.1)	< 0.001	72 (8.3)	73 (8.4)	0.931
Cerebrovascular accident	60 (2.5)	30 (3.4)	0.181	20 (2.3)	27 (3.1)	0.301
COPD	32 (1.3)	21 (2.4)	0.040	16 (1.8)	19 (2.2)	0.608
Renal dysfunction	242 (10.1)	101 (11.4)	0.313	92 (10.6)	98 (11.3)	0.645
Dialysis	13 (0.5)	2 (0.2)	0.381	5 (0.6)	2 (0.2)	0.452
ACS	156 (6.5)	81 (9.1)	0.012	76 (8.8)	76 (8.8)	1.000
Previous PCI	220 (9.2)	162 (18.2)	< 0.001	147 (16.9)	147 (16.9)	1.000
Redo CABG	28 (1.2)	29 (3.3)	< 0.001	23 (2.6)	23 (2.6)	1.000
Emergent operation	93 (3.9)	23 (2.6)	0.071	27 (3.1)	23 (2.6)	0.566
Preoperative laboratory values						
Hb	11.2 (10.1–12.5)	10.9 (9.9–12.2)	0.001	11.1 (10.0-12.4)	11.0 (9.9–12.2)	0.284
Creatinine	0.9 (0.8–1.1)	0.9 (0.8–1.1)	0.939	0.9 (0.8-1.0)	0.9 (0.8–1.1)	0.669
Platelet count	183.3 (150.6-222.4)	191.5 (155.6-235.6)	< 0.001	187.0 (151.8–229.0)	191.4 (155.8-235.2)	0.090
Preoperative ASA	2334 (97.9)	872 (98.1)	0.687	845 (97.4)	838 (96.5)	0.667
Preoperative heparin use	1098 (46.0)	402 (45.2)	0.676	398 (45.9)	394 (45.4)	0.847
Postoperative early use of heparin	251 (10.5)	91 (10.2)	0.811	105 (12.1)	90 (10.4)	0.254
Postoperative anticoagulation	794 (33.3)	296 (33.3)	0.998	302 (34.8)	291 (33.5)	0.578

DM: diabetes mellitus

COPD: chronic obstructive pulmonary disease

ACS: acute coronary syndrome

PCI: percutaneous coronary intervention

CABG: coronary artery bypass grafting

Hb: hemoglobin

ASA: aspirin

### Primary and secondary outcomes in the ASA and DAPT groups after PSM

Differences in the rates of bleeding events in the ASA and DAPT groups after matching are presented in Table 2. Overall, according to the PLATO definitions, major life-threatening bleeding occurred in 16.0% versus 12.8% (*p* = 0.056) of the patients in the ASA and DAPT groups, respectively. Correspondingly, other major bleeding occurred in 26.5% versus 28.6% (*p* = 0.333). Bleeding according to the TIMI major criteria occurred in 6.0% versus 5.5% (*p* = 0.680) and TIMI minor criteria in 22.0% versus 26.4% (*p* = 0.033), in the ASA and DAPT groups, respectively. Similarly, the GUSTO severe bleeding rates were 1.0% and 1.5% (*p* = 0.391). There was no difference in the bleeding-related hemoglobin decrease or reoperation rate between the two groups. Intracranial bleeding was uncommon in both groups. MCTD of > 1 L in the first postoperative hours was seemed to be more frequent in the DAPT group, but the difference was not significant (3.3% vs. 4.8%, *p* = 0.115). Patients in the DAPT group had a higher rate of platelet transfusion, but a lower rate of PRBC transfusion.

**Table 2:** The rates of bleeding events in the ASA and DAPT groups after PSM

	ASA group (*n* = 868)	DAPT group (*n* = 868)	*p*-value
PLATO major bleeding	351 (40.4)	340 (39.2)	0.590
PLATO major life-threatening bleeding	139 (16.0)	111 (12.8)	0.056
PLATO other major bleeding	230 (26.5)	248 (28.6)	0.333
TIMI major bleeding	52 (6.0)	48 (5.5)	0.680
TIMI minor bleeding	191 (22.0)	229 (26.4)	0.033
GUSTO severe bleeding	9 (1.0)	13 (1.5)	0.391
Intracranial bleeding	0 (0.0)	3 (0.3)	0.250
Tamponade	1 (0.1)	1 (0.1)	1.000
Reoperation due to bleeding	9 (1.0)	10 (1.2)	0.818
Bleed resulting in Hb decrease > 50 g/L	52 (6.0)	48 (5.5)	0.680
Bleed resulting in Hb decrease > 30 g/L	236 (27.2)	265 (30.5)	0.125
Transfusion PRBC > 4 U	72 (8.3)	57 (6.6)	0.170
Transfusion PRBC > 2 U	98 (11.3)	68 (7.8)	0.014
PRBC transfusion	129 (14.9)	88 (10.1)	0.003
FFP transfusion	79 (9.1)	82 (9.4)	0.804
Platelet transfusion	43 (5.0)	75 (8.6)	0.002
MCTD > 1 L within 12 h	29 (3.3)	42 (4.8)	0.115

Hb: hemoglobin

PRBC: packed red blood cells

FFP: fresh frozen plasma

MCTD: mediastinal chest tube drainage

The postoperative in-hospital mortality with ASA versus DAPT after matching was 1.5% versus 1.8% (*p* = 0.574) (Table 3). The rate of postoperative MI was higher in the DAPT group 0.2% versus 1.4%, *p* = 0.007). A higher rate was seen with the occurrence of postoperative stroke in the DAPT group, but the difference was not statistically significant (ASA 1.4% versus DAPT 2.2%, *p* = 0.205). The difference in the rate of postoperative MI and stroke drove an increase in the composite endpoint from 3.0% for the ASA group, to 4.0% for the DAPT group, despite the lack of statistical significance (*p* = 0.241).

**Table 3:** The secondary endpoints in the ASA and DAPT groups after PSM

	ASA group (*n* = 868)	DAPT group (*n* = 868)	*p*-value
Postoperative mortality during hospitalization	13 (1.5)	16 (1.8)	0.574
Postoperative in-hospital MI	2 (0.2)	12 (1.4)	0.007
In-hospital Stroke	12 (1.4)	19 (2.2)	0.205
Composite end point	26 (3.0)	35 (4.0)	0.241

MI: myocardial infarction

### Subgroup analysis after PSM

In the ASA group, 566 were treated without anticoagulative therapy (subgroup 1), while 302 were treated with either heparin, LMWH, warfarin or a combination thereof (subgroup 3). Similarly, 577 patients in the DAPT group did not have any anticoagulative therapy (subgroup 2), while 291 received DAPT plus anticoagulative medication (subgroup 4). Heparin and LMWH were used prophylactically in most cases. Warfarin was daily used from 2.5 mg to 5 mg in most cases. No significant difference was seen between subgroup 1 and subgroup 2. Significant increases were seen in the rates of PLATO major life-threatening bleeding and TIMI major bleeding in patients treated with ASA and anticoagulative therapy (subgroup 3) compared with non-anticoagulative ASA treatment (subgroup 1) (Table 4). Patients in subgroup 3 also had higher rates of hemoglobin decrease and PRBC transfusion due to bleeding than subgroup (1) Similarly, patients in subgroup 4 showed higher rates of PLATO major bleeding, TIMI minor bleeding, hemoglobin decrease, PRBC and platelet transfusion due to bleeding than subgroup (2) In addition, according to the surgeon’s prescriptions, a minority of the patients in this study were given heparin within 48 postoperative hours (*n* = 195, 11.2%). They had significantly higher rates of PLATO major bleeding, TIMI major and minor bleeding, bleeding-related hemoglobin decrease and PRBC transfusion (Table 5).

**Table 4:** The rates of bleeding events in the subgroups

	Subgroup 1: ASA (*n* = 566)	Subgroup 2: DAPT (*n* = 577)	Subgroup 3: ASA-anticoag (*n* = 302)	Subgroup 4: DAPT-anticoag (*n* = 291)	*p*-value 1 vs. 2	*p*-value 3 vs. 4	*p*-value 1 vs. 3	*p*-value 2 vs. 4
PLATO major bleeding	196 (34.6)	205 (35.5)	155 (51.3)	135 (46.4)	0.750	0.230	< 0.001	0.002
PLATO major life-threatening bleeding	61 (10.8)	65 (11.3)	78 (25.8)	46 (15.8)	0.792	0.003	< 0.001	0.059
PLATO other major bleeding	142 (25.1)	148 (25.6)	88 (29.1)	100 (34.4)	0.827	0.172	0.198	0.007
TIMI major bleeding	27 (4.8)	31 (5.4)	25 (8.3)	17 (5.8)	0.643	0.248	0.038	0.775
TIMI minor bleeding	116 (20.5)	137 (23.7)	75 (24.8)	92 (31.6)	0.186	0.066	0.142	0.013
GUSTO severe bleeding	5 (0.9)	7 (1.2)	4 (1.3)	6 (2.1)	0.584	0.539	0.508	0.378
Intracranial bleeding	0 (0.0)	1 (0.2)	0 (0.0)	2 (0.7)	1.000	0.240	-	0.262
Reoperation due to bleeding	5 (0.9)	6 (1.0)	4 (1.3)	4 (1.4)	0.786	1.000	0.508	0.739
Bleed resulting in Hb decrease > 50 g/L	27 (4.8)	31 (5.4)	25 (8.3)	17 (5.8)	0.643	0.248	0.038	0.775
Bleed resulting in Hb decrease > 30 g/L	138 (24.4)	163 (28.2)	98 (32.5)	102 (35.1)	0.138	0.503	0.011	0.040
Transfusion PRBC > 4 U	25 (4.4)	31 (5.4)	47 (15.6)	26 (8.9)	0.454	0.014	< 0.001	0.045
Transfusion PRBC > 2 U	40 (7.1)	37 (6.4)	58 (19.2)	31 (10.7)	0.659	0.004	< 0.001	0.028
PRBC transfusion	61 (10.8)	46 (8.0)	68 (22.5)	42 (14.4)	0.104	0.011	< 0.001	0.003
FFP transfusion	44 (7.8)	47 (8.1)	35 (11.6)	35 (12.0)	0.816	0.869	0.063	0.065
Platelet transfusion	29 (5.1)	40 (6.9)	14 (4.6)	35 (12.0)	0.199	0.001	0.752	0.012
MCTD > 1 L within 12 h	20 (3.5)	25 (4.3)	9 (3.0)	17 (5.8)	0.487	0.089	0.666	0.328

ASA: aspirin

DAPT: dual antiplatelet therapy

Hb: hemoglobin

PRBC: packed red blood cells

FFP: fresh frozen plasma

MCTD: mediastinal chest tube drainage

**Table 5:** Early use of heparin and the rates of bleeding events

	Heparin not early used (*n* = 1541)	Heparin early used (*n* = 195)	*p*-value
PLATO major bleeding	576 (37.4)	115 (59.0)	< 0.001
PLATO major life-threatening bleeding	197 (12.8)	53 (27.2)	< 0.001
PLATO other major bleeding	403 (26.2)	75 (38.5)	< 0.001
TIMI major bleeding	79 (5.1)	21 (10.8)	0.001
TIMI minor bleeding	354 (23.0)	66 (33.8)	0.001
GUSTO severe bleeding	19 (1.2)	3 (1.5)	0.730
Intracranial bleeding	2 (0.1)	1 (0.5)	0.301
Reoperation due to bleeding	17 (1.1)	2 (1.0)	1.000
Bleed resulting in Hb decrease > 50 g/L	79 (5.1)	21 (10.8)	0.001
Bleed resulting in Hb decrease > 30 g/L	416 (27.0)	85 (43.6)	< 0.001
Transfusion PRBC > 4 U	98 (6.4)	31 (15.9)	< 0.001
Transfusion PRBC > 2 U	132 (8.6)	34 (17.4)	< 0.001
PRBC transfusion	174 (11.3)	43 (22.1)	< 0.001
FFP transfusion	146 (9.5)	15 (7.7)	0.419
Platelet transfusion	111 (7.2)	7 (3.6)	0.059
MCTD > 1 L within 12 h	68 (4.4)	3 (1.5)	0.056

Hb: hemoglobin

PRBC: packed red blood cells

FFP: fresh frozen plasma

MCTD: mediastinal chest tube drainage

### Multivariable analysis of bleeding-related events and postoperative adverse events after PSM

Logistic regression was performed to ascertain antithrombotic strategies predictive of bleeding-related events and postoperative adverse events (Table 6). Neither preoperative use of ASA nor postoperative use of DAPT was a predictive factor for major or severe bleeding according to PLATO, TIMI or GUSTO. In contrast, postoperative anticoagulant therapy independently predicted PLATO major bleeding (OR: 1.44; 95% CI: 1.14–1.82; *p* = 0.002). Similarly, postoperative use of heparin within 48 h was a predictive factor for PLATO major bleeding (OR: 1.91; 95% CI: 1.34–2.71; *p* < 0.001) and TIMI major bleeding (OR: 2.30; 95% CI: 1.22–4.36; *p* = 0.01). Despite the lack of prediction of major or severe bleeding, postoperative use of DAPT was a predictive factor for TIMI minor bleeding (OR: 1.27; 95%CI: 1.00-1.62; *p* = 0.047).

**Table 6:** Multivariable analysis of bleeding-related events after PSM

	PLATO major		TIMI major		GUSTO severe		TIMI minor		Drainage > 1000mL/12 h		Reoperation	
	OR(95%CI)	*p* value	OR(95%CI)	*p* value	OR(95%CI)	*p* value	OR(95%CI)	*p* value	OR(95%CI)	*p* value	OR(95%CI)	*p* value
Pre. ASA	1.59(0.72–3.51)	0.252	0.79(0.35–1.81)	0.288	0.76(0.10-6.00)	0.998	1.57(0.62–3.97)	0.340	0.59(0.18–2.01)	0.998	0.52(0.06–4.29)	0.998
Pre. DAPT	1.00(0.78–1.29)	0.988	0.68(0.38–1.19)	0.177	1.42(0.53–3.79)	0.481	1.04(0.79–1.38)	0.776	1.88(1.09–3.25)	0.024	2.13(0.75–6.05)	0.156
Post. DAPT	0.97(0.78–1.19)	0.739	1.05(0.68–1.61)	0.833	1.31(0.52–3.30)	0.572	1.27(1.00-1.62)	0.047	1.17(0.69–1.99)	0.566	0.85(0.31–2.33)	0.751
Post. Anticoagulant therapy	1.44(1.14–1.82)	0.002	1.02(0.61–1.70)	0.949	1.68(0.65–4.31)	0.282	1.19(0.92–1.56)	0.191	1.53(0.91–2.57)	0.108	1.55(0.57–4.24)	0.392
Post. heparin early use	1.91(1.34–2.71)	< 0.001	2.30(1.22–4.36)	0.010	0.95(0.24–3.72)	0.937	1.56(1.07–2.28)	0.021	0.27(0.08–0.90)	0.033	0.74(0.15–3.71)	0.711
Redo CABG	1.68(0.93–3.05)	0.087	2.68(1.10–6.57)	0.031	1.74(0.23–13.39)	0.593	1.25(0.65–2.41)	0.512	0.49(0.07–3.62)	0.482	2.02(0.26–15.69)	0.501
Previous PCI	1.19(0.92–1.54)	0.178	0.75(0.41–1.37)	0.350	0.77(0.23–2.61)	0.671	0.94(0.70–1.27)	0.696	0.86(0.44–1.65)	0.641	0.90(0.26–3.12)	0.869

OR: odds ratio

ASA: aspirin

DAPT: dual antiplatelet therapy

CABG: coronary artery bypass grafting

PCI: percutaneous coronary intervention

Postoperative DAPT was associated with higher risk of MI (OR: 6.79; 95% CI: 1.46–31.47; *p* = 0.014); but was not a predictive factor of increased mortality within 30 days (OR: 1.09; 95% CI: 0.49–2.43; *p* = 0.943). Moreover, postoperative use of anticoagulant therapy was significantly associated with increased mortality during hospitalization, a higher risk of in-hospital stroke, and the occurrence of the composite endpoint (Table 7).

**Table 7:** Multivariable analysis of postoperative adverse events after PSM

	Composite end point		in-hostial mortality		MI		Stroke	
	OR(95%CI)	*p* value	OR(95%CI)	*p* value	OR(95%CI)	*p* value	OR(95%CI)	*p* value
Pre. ASA	0.47(0.13–1.68)	0.243	0.75(0.09–6.28)	0.611	0.16(0.03–0.85)	0.031	0.64(0.08–5.14)	0.673
Pre. DAPT	0.67(0.33–1.34)	0.255	1.60(0.67–3.81)	0.026	0.64(0.17–2.45)	0.517	0.40(0.13–1.19)	0.093
Post. DAPT	1.52(0.88–2.62)	0.133	1.09(0.49–2.43)	0.943	6.79(1.46–31.47)	0.014	1.94(0.91–4.14)	0.085
Post. Anticoagulant therapy	3.37(1.89–5.98)	< .001	3.86(1.61–9.26)	0.002	2.85(0.81–9.98)	0.102	3.93(1.84–8.41)	< .001
Post. heparin early use	0.89(0.43–1.83)	0.750	1.38(0.54–3.50)	0.497	1.43(0.36–5.68)	0.612	0.36(0.10–1.28)	0.115
Redo CABG	1.55(0.45–5.28)	0.485	2.40(0.54–10.76)	0.252	0.88(0.21–3.16)	0.745	0.94(0.12–7.23)	0.824
Previous PCI	0.75(0.35–1.59)	0.447	0.57(0.17–1.89)	0.356	0.40(0.05–3.13)	0.384	0.73(0.25–2.10)	0.553

OR: odds ratio

ASA: aspirin

DAPT: dual antiplatelet therapy

CABG: coronary artery bypass grafting

PCI: percutaneous coronary intervention

## Discussion

Antiplatelet therapy is a major strategy to prevent post-CABG failure and adverse events. While aspirin therapy after CABG has been proven to be a safe approach to improve vein graft patency and reduce adverse events [12–14], there have been no dedicated large-scale studies in the CABG population to support postoperative dual antiplatelet therapy. Continuation of DAPT before the surgery increases the risk of excessive perioperative bleeding, transfusions, and re-exploration for bleeding [7, 15, 16], which suggests the likelihood of a high risk of bleeding in postoperative DAPT patients. In a meta-analysis of 20,315 patients, DAPT (as compared to single antiplatelet therapy) was associated with reduced cardiovascular mortality in observational studies, but not in randomized trials or in patients with stable ischemic heart disease. Additionally, DAPT was correlated with an increased risk for major bleeding in that study [17]. The American College of Cardiology/American Heart Association Guideline Focused Update on Duration of Dual Antiplatelet Therapy in Patients with Coronary Artery Disease issued recommendations to utilize DAPT over SAPT in patients following CABG in 2016. Another meta-analysis showed increased vein graft patency with DAPT, but increased postoperative bleeding was also noted [18]. Current guidelines recommend postoperative DAPT based upon the stability of coronary artery disease prior to CABG [2, 19]. In 2017, F. Biancari et al. proposed a Will-Bleed score to evaluate the risk of severe bleeding after CABG [20]. Seven parameters with different weights were included in this model: preoperative anemia, female gender, eGFR, potent antiplatelet drugs discontinued less than five days, critical preoperative state, ACS, and use of LMWH/fondaparinux/unfractionated heparin. This risk score simplified the evaluating process and showed optimistic ability for prediction.

Our study demonstrated that patients who had postoperative DAPT were not at a higher risk for bleeding-related complications than patients on ASA monotherapy. The incidence of major or severe bleeding was balanced between the DAPT and ASA groups, according to the PLATO, TIMI and GUSTO standards. However, TIMI minor bleeding were more often seen in the DAPT group. The multivariable analysis also suggested that postoperative DAPT was not an independent predictor for severe bleeding.

In our study, postoperative DAPT was not associated with a higher rate of perioperative mortality in either univariable or multivariable analysis but was associated with a trend toward an increased risk of postoperative MI and stroke. Since we were unable to know the reason DAPT or ASA was chosen for each patient in the MIMIC database, a possible explanation for these findings is that some patients were given DAPT to treat the onset of postoperative MI or stroke. Additionally, patients in the DAPT group showed a higher percentage of peripheral artery disease, ACS, and previous PCI, possibly indicating a worse status of the artery, which was a reasonable explanation for both the use of DAPT and the higher risks of postoperative MI and stroke. Ticagrelor was regarded as a reasonable substitute for clopidogrel due to its lower risk of CABG-related bleeding in the PLATO trial [7], but it was not evaluated in this study.

Patients undergoing CABG may have an indication for anticoagulants due to various conditions, such as atrial fibrillation, mechanical heart valves, or venous thromboembolism. In some centers, anticoagulants are also used for the prevention of cardiovascular events after a cardiac surgery [6]. The addition of ASA or DAPT to anticoagulant therapy, however, results in at least a two- to threefold increase in bleeding complications [21, 22]. Our study demonstrated that the addition of anticoagulants to either ASA or DAPT increased the risk of bleeding, including the early use of heparin.

We observed the postoperative use of anticoagulants for around one third of the patients. However, we were unable to explore the reason anticoagulant therapy was given to specific patients. Moreover, different types of anticoagulants were not distinguished, though 40.1% of the patients accepting anticoagulant therapy were treated with heparin only, 27.9% with warfarin only, and 32.0% with the combination. Since we failed to acquire the reason for anticoagulant therapy, we cannot conclude that it is inappropriate to add anticoagulants to patients undergoing antiplatelet therapy after the CABG surgery due to the higher risk of bleeding we observed. We believe that it is reasonable to reduce the intensity of anticoagulant therapy or antiplatelet therapy according to the actual situation to lower the bleeding risk.

Several limitations can be seen in this study. First, our study is subject to the limitations of an observational study design including effects from unaccounted confounders and a degree of bias because of potential selection, ascertainment, and treatment-effect bias. It leads to the presence of possible confounding variables that cannot be ruled out completely. Secondly, limited information can be extracted from the database, which led to a large quantity of missing data, and could have led to a mismatch of variables and results. Thus, the multivariable analysis of bleeding-related events and postoperative adverse events is limited by the objective conditions and it cannot provide a comprehensive analysis. We reasoned that the use of DAPT is unlikely to lead to an increased risk of MI, and actual clinical decision-making reflects real practice and most likely would not contradict any randomized controlled trial results. Furthermore, different types of anticoagulants were not separated, as mentioned above, and the percentage of patients with early use of heparin was low. The durations and the onset time of different antithrombotic therapies were also not covered in this study.

We demonstrated that postoperative DAPT was not associated with an increased occurrence of bleeding-related events in patients undergoing isolated CABG and appears to be a safe antiplatelet therapy. In contrast, the addition of anticoagulants to antiplatelet therapy increased the risk of bleeding and should be considered cautiously in clinical practice.

## Data Availability

No datasets were generated or analysed during the current study.

## Abbreviations

ACS: Acute coronary syndrome
ASA: Aspirin
BARC: Bleeding Academic Research Consortium
CABG: Coronary artery bypass grafting
CAD: Coronary artery disease
COPD: Chronic obstructive pulmonary disease
DAPT: Dual antiplatelet therapy
DM: Diabetes mellitus
FFP: Fresh frozen plasma
Hb: Hemoglobin
MCTD: Mediastinal chest tube drainage
MI: Myocardial infarction
OR: Odds ratio
PCI: Percutaneous coronary intervention
PRBC: Packed red blood cells
PSM: Propensity score matching

## References

[1] Neumann FJ, Sousa-Uva M, Ahlsson A (2019). 2018 ESC/EACTS guidelines on myocardial revascularization. Eur Heart J.

[2] Valgimigli M, Bueno H, Byrne RA (2018). 2017 ESC focused update on dual antiplatelet therapy in coronary artery disease developed in collaboration with EACTS: the Task Force for dual antiplatelet therapy in coronary artery disease of the European Society of Cardiology (ESC) and of the European Association for Cardio-thoracic surgery (EACTS). Eur Heart J.

[3] Farooq V, Serruys PW, Bourantas C (2012). Incidence and multivariable correlates of long-term mortality in patients treated with surgical or percutaneous revascularization in the synergy between percutaneous coronary intervention with taxus and cardiac surgery (SYNTAX) trial. Eur Heart J.

[4] Chesebro JH, Clements IP, Fuster V (1982). A platelet-inhibitor-drug trial in coronary-artery bypass operations: benefit of perioperative dipyridamole and aspirin therapy on early postoperative vein-graft patency. N Engl J Med.

[5] Johnson WD, Kayser KL, Hartz AJ, Saedi SF (1992). Aspirin use and survival after coronary bypass surgery. Am Heart J.

[6] Sun JC, Teoh KH, Sheth T (2011). Randomized trial of fondaparinux versus heparin to prevent graft failure after coronary artery bypass grafting: the Fonda CABG study. J Thromb Thrombolysis.

[7] Held C, Asenblad N, Bassand JP (2011). Ticagrelor versus clopidogrel in patients with acute coronary syndromes undergoing coronary artery bypass surgery: results from the PLATO (platelet inhibition and patient outcomes) trial. J Am Coll Cardiol.

[8] Rao AK, Pratt C, Berke A (1988). Thrombolysis in myocardial infarction (TIMI) trial–phase I: hemorrhagic manifestations and changes in plasma fibrinogen and the fibrinolytic system in patients treated with recombinant tissue plasminogen activator and Streptokinase. J Am Coll Cardiol.

[9] Simoons ML, GUSTO IV-ACS investigators (2001). Effect of glycoprotein IIb/IIIa receptor blocker abciximab on outcome in patients with acute coronary syndromes without early coronary revascularisation: the GUSTO IV-ACS randomised trial. Lancet.

[10] Thygesen K, Alpert JS, Jaffe AS (2018). Fourth Universal Definition of Myocardial Infarction (2018). J Am Coll Cardiol.

[11] Mehran R, Rao SV, Bhatt DL (2011). Standardized bleeding definitions for cardiovascular clinical trials: a consensus report from the Bleeding Academic Research Consortium. Circulation.

[12] Ferraris VA, Ferraris SP, Moliterno DJ (2005). The Society of thoracic surgeons practice guideline series: aspirin and other antiplatelet agents during operative coronary revascularization (executive summary). Ann Thorac Surg.

[13] Fremes SE, Levinton C, Naylor CD, Chen E, Christakis GT, Goldman BS (1993). Optimal antithrombotic therapy following aortocoronary bypass: a meta-analysis. Eur J Cardiothorac Surg.

[14] Dunning J, Versteegh M, Fabbri A (2008). Guideline on antiplatelet and anticoagulation management in cardiac surgery. Eur J Cardiothorac Surg.

[15] Fox KA, Mehta SR, Peters R (2004). Benefits and risks of the combination of clopidogrel and aspirin in patients undergoing surgical revascularization for non-ST-elevation acute coronary syndrome: the Clopidogrel in unstable angina to prevent recurrent ischemic events (CURE) Trial. Circulation.

[16] Smith PK, Goodnough LT, Levy JH (2012). Mortality benefit with prasugrel in the TRITON-TIMI 38 coronary artery bypass grafting cohort: risk-adjusted retrospective data analysis. J Am Coll Cardiol.

[17] Cardoso R, Knijik L, Whelton SP (2018). Dual versus single antiplatelet therapy after coronary artery bypass graft surgery: an updated meta-analysis. Int J Cardiol.

[18] Deo SV, Dunlay SM, Shah IK (2013). Dual anti-platelet therapy after coronary artery bypass grafting: is there any benefit? A systematic review and meta-analysis. J Card Surg.

[19] Levine GN, Bates ER, Bittl JA (2016). 2016 ACC/AHA Guideline focused update on duration of dual antiplatelet therapy in patients with coronary artery disease: a report of the American College of Cardiology/American Heart Association Task Force on clinical practice guidelines: an update of the 2011 ACCF/AHA/SCAI guideline for percutaneous coronary intervention, 2011 ACCF/AHA guideline for coronary artery bypass graft surgery, 2012 ACC/AHA/ACP/AATS/PCNA/SCAI/STS guideline for the diagnosis and management of patients with stable ischemic heart disease, 2013 ACCF/AHA guideline for the management of ST-Elevation myocardial infarction, 2014 AHA/ACC guideline for the management of patients with Non-ST-Elevation acute coronary syndromes, and 2014 ACC/AHA guideline on perioperative cardiovascular evaluation and management of patients undergoing noncardiac surgery. Circulation.

[20] Biancari F, Brascia D, Onorati F (2017). Prediction of severe bleeding after coronary surgery: the WILL-BLEED risk score. Thromb Haemost.

[21] sorensen R, Hansen ML, Abildstrom SZ (2009). Risk of bleeding in patients with acute myocardial infarction treated with different combinations of aspirin, clopidogrel, and vitamin K antagonists in Denmark: a retrospective analysis of nationwide registry data. Lancet.

[22] Dans AL, Connolly SJ, Wallentin L (2013). Concomitant use of antiplatelet therapy with dabigatran or warfarin in the Randomized evaluation of long-term anticoagulation therapy (RE-LY) trial. Circulation.

